# Food Addiction and Emotional Eating Behaviors Co-Occurring with Problematic Smartphone Use in Adolescents?

**DOI:** 10.3390/ijerph19094939

**Published:** 2022-04-19

**Authors:** Eun-Jin Park, Samuel Suk-Hyun Hwang, Mi-Sun Lee, Soo-Young Bhang

**Affiliations:** 1 Department of Psychiatry, Ilsan Paik Hospital, College of Medicine, Inje University, Goyang 10380, Korea; i0277@paik.ac.kr; 2 Center for School Mental Health, Eulji University, Seoul 01830, Korea; dlaltjs1010@hanmail.net; 3 Department of Psychology, Chonnam National University, Gwangju 61186, Korea; hwansama@hanmail.net; 4 Department of Preventive Medicine, College of Medicine, The Catholic University, Seoul 06591, Korea; 5 Department of Psychiatry, Eulji University Hospital, School of Medicine, Eulji University, Seoul 01830, Korea

**Keywords:** food addiction, problematic smartphone use, eating behavior, adolescents

## Abstract

Addiction in adolescence is increasing and has a significant impact on physical and mental health. Notably, addictions can be comorbid and affect each other. Despite the recent growing interest in food addiction (FA) and problematic smartphone use (PSU), few studies have investigated their association in adolescents. We investigated the relationship between FA and PSU in adolescents and the effects of eating behaviors. A total of 209 adolescents (44.5% male; mean age = 12.86 ± 0.7 years) participated in the current school-based community study. We found a positive correlation between the dimensional Yale Food Addiction Scale for Children 2.0 (dYFAS-C2.0) and the Smartphone Overdependence Scale after adjusting for age, sex, body mass index, and socioeconomic status. The high-risk PSU group accounted for 17.2% of participants. Furthermore, this group showed 2.3 times higher dYFAS-C2.0 scores than the general group. Emotional overeating and satiety responsiveness were correlated with PSU. A comprehensive evaluation of addiction symptoms is needed for proper intervention, especially in adolescents with symptoms of abnormal eating behaviors.

## 1. Introduction

Physical and mental health are critical for the normal development of adolescents. Healthy eating habits and controlled multimedia use are essential for maintaining good health and functioning. Food addiction (FA) is defined by an uncontrollable urge to eat sugary and fatty food [1]. Studies on FA are increasing with the growing interest in nutrient-rich diets, especially in children and adolescents. FA is associated with obesity and eating disorders [2] and can lead to physical health problems such as hypertension and diabetes [3]. In addition, FA may increase depression, anxiety, emotional dysregulation, and impulsivity [2,4,5,6]. Therefore, FA has been negatively associated with poor academic performance [7] and quality of life [6].

Previous studies have reported high incidences of FA in adolescents and women who are obese or have eating disorders, diabetes, or schizophrenia [8,9,10]. According to a large-scale study, the prevalence of FA in adolescents was reported to be 2.6–6.9% [6,11]; however, FA has been found to differ depending on the sample, sex, comorbidities, and region. In community samples, the prevalence of FA varies between 4–10%, with the prevalence increasing up to 26.2% in obese women and 42.3% in participants with binge eating disorders [8,11,12,13,14]. In particular, FA is related to eating behaviors and psychological conditions that affect other addictions in adolescents which can be comorbid and affect each other. In an extensive study of teenagers, FA was linked to smoking, alcohol consumption, and cannabis use, demonstrating that several addictions can co-occur [11].

The widespread use of smartphones has also affected the daily life of adolescents. Smartphones can be used for a wide range of tasks, including accessing the Internet, communicating with others, having fun, and even keeping track of one’s health. With portability, real-time connectedness and seemingly limitless app options, smartphones have become an indispensable part of our daily lives [15].

As media and technology use has increased, research interest in problematic internet and smartphone use has also increased [16,17]. Numerous attempts have been made to explain adolescents’ excessive use of the internet and validate the phenomenon’s definition, concept, and characteristics [18,19]. Recently, DSM-5 section III and ICD-11 have defined internet gaming disorder criteria and gaming disorder criteria, respectively. Although the terms are mixed in recent research, problematic internet use (PIU) is considered an addiction in terms of tolerance, withdrawal symptoms, and loss of control. PIU refers to excessive or compulsive internet use, which leads to loss of control and spending too much time on the internet. As a result, functional impairment occurs in areas such as social relationships, health, academic skills, and sleep [20].

According to a study reviewing the concepts of internet use (IU) and problematic internet use (PIU) on the continuum of behavior, PIU negatively affects daily functioning and interpersonal and emotional health. In addition, these symptoms are similar to substance-related addiction [21]. With the development of various media, the concept of problematic interactive media use (PIMU) may explain the phenomenon more appropriately [22]. PIMU broadens the definition of media usage, implying excessive use of gaming, social media, and information retrieval [22]. The concept of problematic smartphone use (PSU) focuses on the use of smartphones and is similar to PIMU. PSU is a relatively new concept and is not clearly defined. Traditionally, addiction refers to a state of loss of control even when negative consequences are expected from using certain substances and has been described in terms of substance and behavioral addictions. PSU can be viewed as a continuum of PIU or PIMU and is defined as the continuous and excessive use of smartphones to access various internet content due to the development of specific technologies.

PSU is particularly common among adolescents, ranging from 2–20% [23,24] globally. The Korean government conducts an annual survey on internet and smartphone use among adolescents. Problematic internet and smartphone use has been collectively defined as the term “smartphone overdependence”. The diagnostic criteria for ICD-10 codes are contained in the survey questionnaire. Smartphone overdependence is characterized as ‘excessive use of the internet and smartphones that results in tolerance, withdrawal symptoms, and impairment in daily life’ [25]. According to the results of the annual survey, 37% of Korean adolescents aged 10–19 were at risk of smartphone overdependence in 2021 [26]. In adolescents, PSU has been demonstrated to lead to hypertension and neck disability, while severe addiction increases depression and impulse control disorders [23,24,27].

Understanding addiction in adolescents is essential given the importance of brain development. High levels of novelty-seeking and risk-taking can characterize their behaviors. Importantly, exposure to an unfavorable social environment is likely to harm emotional processing and brain plasticity [28]. Therefore, teenagers with such features may be at high risk of addiction [11].

Addictions can have significant adverse effects on neurodevelopment. FA and PSU are relatively common among adolescents and can be related to each other. A recent systematic review suggested that PIU may be a predictor of eating disorders, and adolescent smartphone addiction can be associated with maladaptive eating behaviors [29,30]. Despite the growing interest in adolescent addiction and vulnerability, there is little research on the direct relationship between FA and PSU. Therefore, the purpose of the present study was to elucidate the relationship between FA and PSU and investigate the risk factors influencing their severity.

## 2. Materials and Methods

### 2.1. Participants and Procedure

The study was conducted as part of a school-based mental health promotion program. We sent letters to schools to solicit participation, and three middle schools indicated interest. The program was designed to prevent mental health conditions such as depression and to promote healthy lifestyles among students. The program included modifying lifestyles to decrease the use of smartphones, achieve an adequate amount of sleep, maintain healthy eating habits by limiting junk food, and engage in outdoor exercise.

To increase mental health awareness, we evaluated FA, smartphone usage, and dietary habits among the participants. Of the 310 adolescents who participated, 209 completed all questionnaires and were included in the analysis. The questionnaires assessed age, sex, height, weight, socioeconomic status (SES), exercise time, multimedia usage time, binge eating, and emotional overeating.

### 2.2. Measures

#### 2.2.1. The Dimensional Yale Food Addiction Scale for Children 2.0

The dimensional Yale Food Addiction Scale for Children 2.0 (dYFAS-C2.0) consists of 16 items and is a self-administered scale to assess symptoms of FA among adolescents. The dYFAS-C2.0 was revised to reflect the updated diagnostic approaches for addictive disorders [31]. The dYFAS-C2.0 has demonstrated good validity and reliability and has been positively associated with body mass index (BMI) and emotional eating. The present study administered the Korean version of the dYFAS-C2.0 to the participants.

After obtaining permission from the original author, Korean-style junk food was added according to a recent study by the Korean National Health and Nutrition Examination Survey. This included street food (e.g., spicy rice cakes, fried foods, pork blood sausage, and fish cakes), instant food products (e.g., instant noodles, frozen foods, and retort pouch foods [sealed and sterilized food sold in pouches]), and deliverable fast foods (e.g., fried chicken and jajangmyeon [Korean black-bean-sauce noodles]).

#### 2.2.2. Korean Smartphone Overdependence Scale for Adolescents

Adolescent problematic smartphone use was assessed using the Korean-language Smartphone Overdependence Scale. This questionnaire comprises ten items related to loss of control, pervasiveness, and adverse consequences. The scale was self-reported using a 4-point Likert scale. Based on the Smartphone Addiction Proneness Scale, which was developed in 2011 [32], and was reorganized in 2014, the Korean Smartphone Overdependence Scale was developed to screen for PSU in 2016. The survey contents include the following. “I fail to reduce the smartphone use time”, “it is difficult to control the time spent using the smartphone”,” it is difficult to keep the proper smartphone usage time”, “it is difficult to focus on other things when a smartphone is next to me”, “I’m constantly reminded of my smartphone”, “I feel a strong urge to use my smartphone”, “I have had health problems due to smartphone use”, “I had an argument with my family over my smartphone”, “I have experienced severe conflicts with friends, colleagues, and social relationships because of the use of smartphone”, and “I have difficulties with work (study, work, etc.) due to a smartphone”.

The participants were categorized into three groups based on their total scores, with higher scores demonstrating higher levels of smartphone overdependence. Cutoff scores vary based on age. Therefore, adolescent participants were classified into one of three groups: high risk (≥31 points), potential risk (23–30 points), and general (≤22 points). In an additional analysis, participants were divided into two groups and the relationships between eating behaviors, FA, and other variables were compared (group without risk, ≤22 points; group with risk, ≥23 points).

#### 2.2.3. Child Eating Behavior Questionnaire

The Child Eating Behavior Questionnaire (CEBQ) is a brief parent-reported measure of children’s eating behavior traits that is widely used in research and clinical settings. The CEBQ was developed and validated in the United Kingdom and is one of the most comprehensive scales for assessing children’s eating behaviors. The CEBQ in the present study consists of 10 items on a 5-point Likert scale (never, rarely, sometimes, often, and always) for assessing eating behaviors. The Korean version of the CEBQ has been previously translated and validated [33]. Higher scores indicate a greater frequency of eating behavior on each subscale. The present study reported data on two subscales: emotional overeating and satiety responsiveness [34]. Emotional eating and satiety responsiveness are defined as the tendency to overeat in response to negative or positive emotions and the ability to regulate food intake in relation to satiety, respectively.

### 2.3. Statistical Analysis

All data analyses were performed using SPSS (version 25.0; IBM, Armonk, NY, USA). Descriptive statistics are presented as mean ± standard deviation or as numbers and percentages for categorical data. A chi-squared test was performed to determine the relationships between categorical variables. Partial correlation analyses were conducted after adjusting for covariates. Multiple linear regressions and analyses of covariance were used to evaluate the differences in risk groups after adjusting for age, sex, SES, and BMI percentiles. Logistic regression analysis was conducted to determine risk factors for PSU. The results were considered statistically significant at *p* < 0.05.

### 2.4. Ethics

The study procedures were performed in accordance with the Declaration of Helsinki. The participants voluntarily completed the questionnaires and the study was approved by the institutional review board (no. 2018-12-011).

## 3. Results

A total of 209 students (male = 93, 44.5%) with an average age of 12.86 (±0.70) years were analyzed. The mean dYFAS-C2.0 and Smartphone overdependence scale scores were 8.5 (±7.5) and 22.6 (±8.5) points, respectively. There was no difference in age between sexes (*p* = 0.064). When calculated as BMI percentile, 4.3% of participants were in the obese group (≥95th BMI percentile). The dYFAS-C2.0 scores were significantly higher in the high BMI group (BMI above the 90th percentile; *p* = 0.021, adjusted for age, sex, and SES).

There were significant differences in BMI percentages between boys and girls. The average BMI percentile was lower in girls than boys (*p* = 0.001). For BMI in girls, 8.6% were above the 90th percentile and 26.7% were below the 10th percentile. In comparison, 18.3% of boys were above the 90th percentile and 16.1% were below the 10th percentile.

In the assessment of eating behaviors, uncontrolled eating experiences were not significantly different between boys and girls (*p* = 0.777), but binge eating experiences were significantly more frequent in boys (*p* = 0.013). Girls also showed a higher tendency for emotional overeating (*p* = 0.001) and satiety responsiveness (*p* < 0.001). Table 1 summarizes the results.

### 3.1. Relationship between dYFAS-C2.0 and PSU

**Figure 1:**
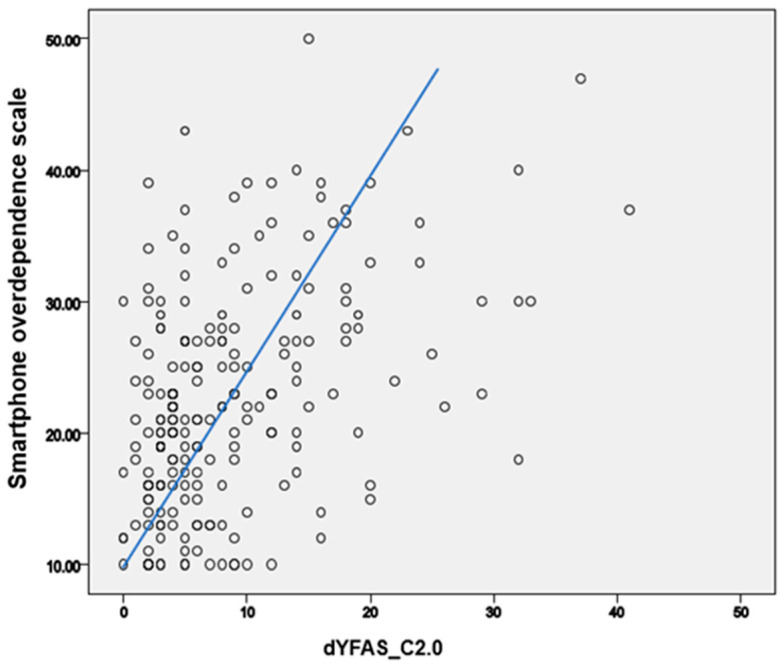
Correlation between food addiction and problematic smartphone use.

The correlation between the dYFAS-C2.0 and Smartphone Overdependence Scale scores was analyzed. The dYFAS-C2.0 and Smartphone Overdependence Scale scores were significantly correlated after adjusting for age, sex, BMI percentile, and SES (r = 0.428, *p* < 0.001; Figure 1). This relationship remained significant after multiple linear regression analyses (age, sex, BMI percentile, and SES were corrected; B = 0.426, *p* < 0.001).

**Figure 2:**
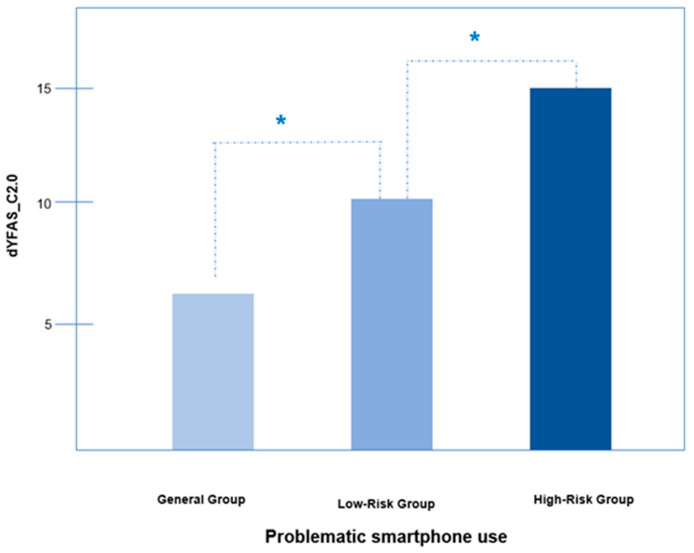
Differences of food addiction by problematic smartphone use severity. Abbreviation: dYFAS_C2.0 scale: dimensional Yale Food Addiction Scale for Children 2.0. *: *p*-value < 0.05. In the case of high-risk smartphone addiction, food addiction also increased. and the difference was significant for each group when adjusting for age, gender, socioeconomic status (SES), and body mass index (BMI) percentile (F = 13.419, *p* < 0.001).

We compared dYFAS-C2.0 scores of participants divided into three groups based on PSU scores (i.e., general, low-risk, and high-risk groups). Groups (general, low-risk, and high-risk) consisted of 113 (54.1%), 60 (28.7%), and 36 (17.2%) participants, respectively, with dYFAS-C2.0 scores of 6.1 (±5.3), 10.0 (±8.0) and 14.4 (±9.2) points, respectively. The dYFAS-C2.0 score of the high-risk group was approximately 2.3 times higher than that of the general group. The difference was significant for each group after adjusting for age, sex, SES, and BMI percentile (F = 13.419, *p* < 0.001; Figure 2).

### 3.2. Sedentary Lifestyle and Food Addiction, and PSU

The time spent using multimedia and average daily exercise time were not related to the severity of the dYFAS-C2.0 scores (*p* = 0.122 and *p* = 0.294, respectively). Multimedia use time was related to the Smartphone Overdependence Scale score (F = 37.070, *p* < 0.001), but daily exercise time was not (F = 3.376, *p* = 0.068; Table 1).

### 3.3. Eating Behaviors, FA, and PSU

Binge eating was significantly associated with dYFAS-C2.0 scores (r = 0.272, *p* < 0.001) and uncontrolled eating (r = 0.261, *p* < 0.001). Additionally, emotional overeating was significantly correlated with dYFAS-C2.0 (r = 0.438, *p* < 0.001) and PSU scores (r = 0.238, *p* < 0.01). Satiety responsiveness was not correlated with the dYFAS-C2.0 scores (*p* = 0.217); however, it was correlated with PSU scores (r = 0.158, *p* < 0.05). Table 2 showed the characteristics of eating problems according to the risk of smartphone use. The high-risk group for problematic smartphone use showed relatively higher FA scores (*p* < 0.001), emotional overeating behavior (*p* = 0.001), and satiety responsiveness (*p* = 0.041).

### 3.4. Effect of Eating Behaviors and FA on PSU

Logistic regression analysis was conducted after adjusting for sex, age, BMI percentiles, and SES. The dYFAS-C2.0 score (*p* = 0.003), emotional overeating (*p* = 0.016), and satiety responsiveness (*p* = 0.015) increased the risk of PSU (see Table 3).

## 4. Discussion

The present study investigated the relationship between FA and PSU in adolescents using a school-based community sample. When the severity of PSU increased, the level of FA also increased. The dYFAS-C2.0 score was significantly higher in the high BMI group. Binge eating, uncontrolled eating experiences and emotional overeating were significantly associated with the dYFAS-C2.0 score. Additionally, emotional overeating and satiety responsiveness were positively correlated with PSU. There was no difference in dYFAS-C2.0 and PSU scores based on sex.

We explored the significant and positive correlation between FA severity and PSU. The high-risk for the PSU group’s dYFAS-C2.0 score was approximately 2.3 times that of the general group (6.1 ± 5.3 versus 14.4 ± 9.2; Figure 2). Few studies have directly investigated FA and PSU; however, several studies have inferred their association. Overuse of social network services (SNS) co-occurs with FA, which is associated with symptoms of internet-use disorders [35,36]. Other researchers have reported that internet addiction is associated with fast food consumption, and using smartphones during meals may lead to increased caloric intake [37].

Our study suggests that FA and smartphone addiction may co-occur in adolescents. One addiction can raise the risk of developing another addiction. A survey of college students revealed that the sharing behaviors on social media were similar to self-promotion and peer promotion of alcohol use [38]. Gaming disorder can co-occur with a variety of other addictive behaviors such as alcohol use and the addictive use of social media [39]. Research investigating the co-occurrence of addictive behaviors and substance use is increasing. Our study also showed an association between FA and PSU and the potential for concomitant addiction.

Reports on comorbid addictions have become increasingly common, and impairments in decision-making in PSU are similar to those found in alcohol, gambling, and spending addictions [40]. In a study on FA in gambling disorders, participants with high FA scores showed more severe psychopathologies [41]. Moreover, various addictions have been found to share cognitive characteristics. Nolan and Jenkins (2019) suggested that irrational beliefs were significantly correlated with FA, and that elevated anxiety and depression due to such beliefs intensified FA [42].

FA and PSU in adolescents may have similar neural mechanisms in which dopamine and reward networks have essential roles [43,44,45]. Recent studies have reported that the risk of FA increases dopamine receptor D2 levels in the nucleus accumbens [46]. Moreover, a functional magnetic resonance imaging study showed altered response inhibition and error processing in FA [47]. Smartphone addiction is significantly correlated with dopamine transport levels [48]. These studies imply that high-calorie foods and smartphone overuse may activate the brain’s reward system to release dopamine.

Problematic eating behavior affected FA, and PSU was also associated with eating behaviors in our study. In addition, our results showed that PSU was considerably correlated with emotional overeating and satiety responsiveness. A logistic regression analysis showed that the odds ratio of eating behaviors increased to predict the risk of PSU (odds ratios of FA, emotional overeating, satiety responsiveness: 1.111 [1.045–1.180], 1.119 [1.009–1.240], and 1.117 [1.013–1.232], respectively). A recent study reported a link between eating habits and PSU in adolescents, demonstrating that adolescents with PSU showed different eating patterns, including a higher frequency of skipped meals [49]. Adolescents in the high addiction group also consumed fewer nutrient-dense foods, suggesting a link between unhealthy eating patterns and PSU [49]. In the current study, emotional overeating was found to be related to PSU. Emotional overeating often occurs in response to stress, boredom, unhappiness, or other emotional conditions rather than physical hunger. Emotional eating is associated with depression [50], and it mediates the effect of depression on BMI [51]. Continued emotional distress can lead to depression, contributing to abnormal eating behaviors, such as emotional eating, which subsequently increases the risk of PSU and FA. In contrast, we showed that higher satiety responsiveness was associated with higher PSU. Previous studies have demonstrated that poor satiety response is a risk factor for overeating [52]. Therefore, a larger group investigation of satiety responsiveness is required.

Our research is consistent with previous findings linking FA to obesity and abnormal eating behaviors. In previous studies, the prevalence of FA was found to be higher in obese women and those with binge eating disorder [14,53,54,55]. Our results demonstrated that the dYFAS-C2.0 was positively associated with emotional and binge eating, with higher scores observed in the obese group, which is consistent with previous investigations. Additionally, body image dissatisfaction, related to problematic smartphone use among adolescents, is likely connected to obesity and FA because body uneasiness is independently associated with FA symptoms [56,57]. Moreover, a recent meta-analysis showed that problematic internet use could predict eating disorders [29]. Consistent with our results and considering that social media and internet use can increase smartphone use and that high-calorie eating habits are linked to FA, these studies suggest a close relationship between FA and PSU.

FA and PSU share similar psychopathologies such as depression, anxiety, sleep disturbances, and impulsivity [58,59]. Along with poor self-esteem and body image dissatisfaction, depression is the most common emotional difficulty associated with FA [55,60,61]. A recent study showed that increased duration of smartphone usage increases the risk of depressive symptoms with odds ratios of 1.18 (1.10–1.26) [62]. Excessive smartphone use is associated with social isolation, decreased self-control, and higher daily life stress [63,64]. High-stress levels also increase the risk of PSU [65]. Furthermore, loneliness is a strong predictor of PSU, while high self-esteem may be a protective factor against PSU in adolescents [66,67]. Moreover, through depression, maladaptive metacognition influenced smartphone addiction indirectly [68]. These findings suggest that adolescents’ lack of social networks may deprive them of feelings of support and comfort from social interactions in an offline environment, which can intensify their desire to be absorbed by their smartphones [69]. Adolescents with FA or PSU require extensive evaluation of emotional symptoms for appropriate interventions.

Conversely, attempts to correct addictive behavior using digital therapeutics are also increasing. According to research on digital nudges for reducing social media addiction, digital nudges are intended to help individuals gain a more objective view of their social media use, control their usage time, and have a more pleasant experience [70]. Additionally, with the current surge in behavioral therapy attempts via smartphone apps, the usage of smartphones as a treatment tool is demonstrating promise [71]. Moreover, monitoring symptoms of eating disorders via an app on a smartphone is helpful [72]. Such apps can also be used for cognitive behavioral therapy for addiction treatment [73] or obtaining self-help [74]. We should also emphasize the role that digital platforms can play in promoting positive behavior. In relation to FA, both the risk and applicability of smartphone use should be studied further in the future.

Our study examined sex differences across various areas. The dYFAS-C2.0 and Smartphone overdependence scale scores did not differ by sex; however, a higher percentage of girls were in the high-risk group for PSU (girls: 63.5%, *p* = 0.022). Previous findings on sex differences in FA and PSU are controversial. Some studies have indicated that FA is more common in women [2,10,75], whereas others have reported no sex differences [76]. Regarding PSU, there are studies that show no sex difference [23,77], and some studies show that it is more common in women [78,79,80]. In the present study, girls exercised less, spent more time on multimedia, and showed more eating problems; however, their overall BMI was lower than boys. Boys were more likely to engage in binge eating, while girls were more likely to engage in emotional overeating. Girls showed higher satiety responsiveness than boys. These results are similar to previous studies that have reported more problematic eating behaviors in women. Our results showed that boys had more binge eating experiences; however, a previous study found that the rate was higher in girls, which was also confirmed in adults [81,82]. In a study on binge eating in boys, binge eating was significantly associated with age, BMI, experience-seeking tendency, and boredom susceptibility [81]. Furthermore, recent evidence indicates that eating disorders are not uncommon in men and are equally severe in symptom presentation [83]. Further studies on men’s eating behaviors are needed.

Our current study has several limitations. The primary limitation is that it was a cross-sectional study involving a community sample. Second, a small sample size was used. Future studies with larger samples are required to better understand the relationship between FA and PSU. Participant height and weight were not directly measured; rather, these were self-reported, which should be considered when interpreting our results. Further studies are needed using larger samples and systematic diagnostic evaluations in the future.

## 5. Conclusions

This study investigated the relationship between FA and PSU in adolescents using the dYFAS-C2.0. Results showed that the severity of PSU was associated with FA. FA and PSU in adolescents were found to be related to unhealthy eating and a sedentary lifestyle. Moreover, PSU and FA in adolescents were more associated with shared mental health conditions and common biological reward mechanisms of addiction. High-calorie and high sugar foods may also trigger and reinforce addictive behaviors that intensify emotional difficulties. For the healthy development of adolescents, it is critical to prioritize physical activity, appropriate media use, and healthy dietary behaviors, as well as to develop a variety of programs and opportunities for daily support. A comprehensive evaluation of addiction symptoms is necessary, especially for adolescents with symptoms of unhealthy eating behaviors.

## Figures and Tables

**Table 1:** Demographic characteristics of participants.

	Total	Boys	Girls	*t*-Test	x^2^ Test
	*n* = 209	93 (44.5%)	116 (55.5%)		
Age	12.8 (±0.7)	12.6 ± 0.5	12.9 ± 0.7	*p* = 0.064	
BMI percentile	39.2 ± 29.9	47.9 ± 31.7	32.6 ± 26.8	*p* = 0.001	
≤5th	14 (6.7%)	4 (4.3%)	30 (8.6%)		*p* = 0.019
5th–10th	11 (11.8%)	11 (11.8%)	21 (18.1%)		
10th–90th	61 (65.6%)	61 (65.6%)	75 (64.7%)		
90th–95th	13 (14.0%)	13 (14.0%)	6 (5.2%)		
≥95th	4 (4.3%)	4 (4.3%)	4 (3.4%)		
SES					*p* = 0.771
low to moderate	22 (10.6%)	11 (12.1%)	11 (10.6%)		
moderate	130 (62.8%)	55 (60.4%)	75 (62.8%)		
moderate to high	55 (26.6%)	25 (27.5%)	30 (26.6%)		
Exercise time					
less than 1 h/day	116 (55.8%)	36 (39.1%)	80 (69%)		*p* < 0.001
more than 1 h/day	92 (44.2%)	56 (60.9%)	36 (31%)		
Multimedia use time					
less than 2 h/day	96 (46.2%)	50 (54.3%)	46 (39.7%)		*p* = 0.037
more than 2 h/day	112 (53.8%)	42 (45.7%)	70 (60.3%)		
Binge eating experiences					
No	101 (48.6%)	37 (40.2%)	64 (55.2%)		*p* = 0.037
Yes	107 (51.4%)	55 (59.8%)	52 (44.8%)		
Uncontrolled eating experiences					
No	138 (66.3%)	55 (59.8%)	66 (56.9%)		*p* = 0.777
Yes	87 (33.7%)	37 (40.2%)	50 (43.1%)		
CEBQ_EOE	10.4 ± 4.7	9.3 ± 3.9	11.2 ± 4.1	*p* = 0.001	
CEBQ_SR	12.3 ± 3.9	10.7 ± 3.6	13.6 ± 3.7	*p* < 0.001	
dYFAS-C2.0	8.7 ± 7.5	8.3 ± 7.6	9.0 ± 7.5	*p* = 0.519	
Smartphone Overdependence Scale	22.6 ± 8.5	21.4 ± 8.1	23.6 ± 8.7	*p* = 0.061	

Abbreviations: BMI, body mass index; SES, socioeconomic status; CEBQ_EOE, Child Eating Behavior Questionnaire Emotional Overeating; CEBQ_SR, Child Eating Behavior Questionnaire Satiety Responsiveness; dYFAS_C2.0, dimensional Yale Food Addiction Scale for Children 2.0.

**Table 2:** The comparison between eating behaviors and food addiction in a group with problematic smartphone use risk.

	Total	Problematic Smartphone Use		
		Group without Risk	Group with Risk	
	*n* = 209	*n* = 113	*n* = 96	*p* Value
Age (years)	12.86 (±0.70)	12.8 ± 0.6	12.9 ± 0.9	0.149
Girls (%)	93 (44.5%)	55 (48.7%)	61 (63.5%)	0.022
BMI percentile	39.27 ± 29.94	43.1 ± 30.4	34.8 ± 28.9	0.074
CEBQ_EOE	10.40 ± 4.17	9.4 ± 3.9	11.4 ± 4.1	0.001
CEBQ_SR	12.36 ± 3.98	11.8 ± 4.0	12.9 ± 3.8	0.041
dYFAS_C2.0	8.7 ± 7.5	6.1 ± 5.3	11.7 ± 8.7	<0.001
Smartphone Overdependence Scale	22.63 ± 8.52	16.2 ± 3.9	30.1 ± 5.9	<0.001

Abbreviations: BMI, body mass index; SES, socioeconomic status; CEBQ_EOE, Child Eating Behavior Questionnaire Emotional Overeating; CEBQ_SR, Child Eating Behavior Questionnaire Satiety Responsiveness; dYFAS_C2.0, dimensional Yale Food Addiction Scale for Children 2.0.

**Table 3:** Logistic regression analysis of food addiction and eating behaviors on problematic smartphone use.

						95% Confident Interval	
	B	S.E.	F	*p*-Value	Exp(B)	Lowest	Highest
dYFAS_C2.0	0.105	0.031	1	0.001	1.111	1.045	1.180
CEBQ_EOE	0.112	0.052	1	0.032	1.119	1.009	1.240
CEBQ_SR	0.111	0.050	1	0.027	1.117	1.013	1.232

Logistic regression analysis was conducted after adjusting for gender, age, BMI percentile, and SES. Abbreviations: BMI, body mass index; SES, socioeconomic status; CEBQ_EOE, Child Eating Behavior Questionnaire Emotional Overeating, CEBQ_SR: Child Eating Behavior Questionnaire Satiety Responsiveness; dYFAS_C2.0, dimensional Yale Food Addiction Scale for Children 2.0.

## Data Availability

The data that support the findings of this study are available from the corresponding author upon reasonable request.
